# Porous carbon adsorption layer enabling highly reversible redox-reaction of a high potential organic electrode material for sodium ion batteries†

**DOI:** 10.1039/c8ra03093f

**Published:** 2018-07-11

**Authors:** Yanjie Wang, Chun Fang, Ying Huang, Qing Liu, Ruirui Zhao, Xuli Ding, Yunhui Huang

**Affiliations:** Collaborative Innovation Center of Intelligent New Energy Vehicle, School of Materials Science and Engineering, Tongji University Shanghai 201804 China huangyh@tongji.edu.cn; State Key Laboratory of Material Processing and Die & Mould Technology, School of Materials Science and Engineering, Huazhong University of Science and Technology Wuhan Hubei 430074 China fangchun@hust.edu.cn; School of Materials Science and Engineering, Yunnan Key Laboratory for Micro/Nano Materials & Technology, Yunnan University Kunming Yunnan 650091 China; Department of Physics, Jiangsu University of Science and Technology Zhenjiang Jiangsu 212003 China

## Abstract

Organic compounds have been utilized in rechargeable batteries as electrode materials on account of their designable structures and reversible redox properties. However, most of them suffer from problems with dissolution resulting in poor electrochemical performance. In this work, we adapt a sodium salt of tetracyanoquinodimethane (NaTCNQ) to work as a high redox potential cathode material in sodium ion batteries (SIBs). A porous carbon coated separator is demonstrated to be an adsorption layer and prevents the dissolved active material from migrating to the anode side. The NaTCNQ cell assembled with a carbon layer containing 5% activated carbon (AC) exhibits a higher initial capacity and greatly improved cycling stability. Using a conductive adsorption layer in organic redox batteries is a promising pathway to develop high performance organic electrode materials for SIBs.

## Abstract 2

A highly reversible organic cathode material is stabilized by preventing the dissolved active material from shuttling using a porous carbon layer.

## Graphical abstract

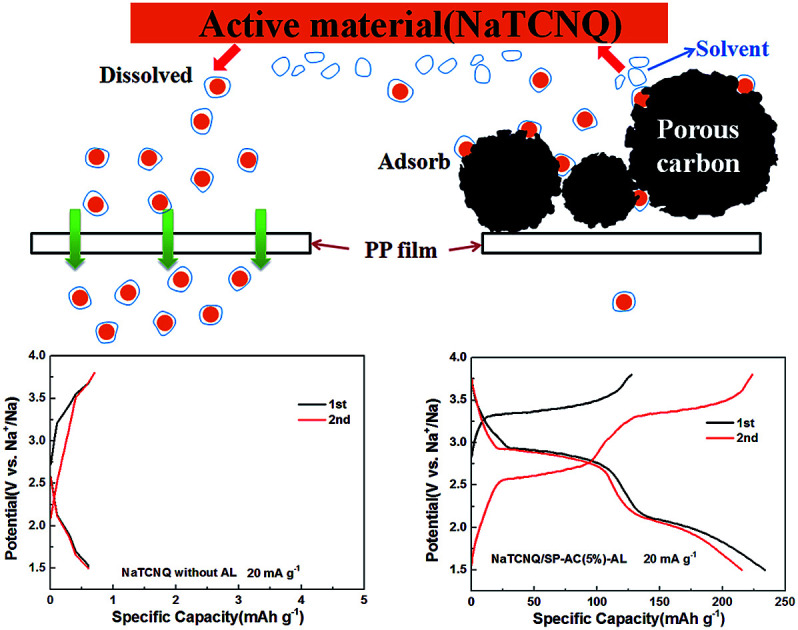

## Introduction

Nowadays, sodium-ion batteries (SIBs) have attracted more and more attention as lithium-ion batteries (LIBs) are facing some seemingly insurmountable, relatively long-term problems. In particular, the scarcity of lithium resources has promoted the rapid development of sodium ion batteries.^1,2^ Advantages such as abundant resources, low price and environmental friendliness mean that SIBs have enormous potential for application in energy storage.^3–5^ However, most studies are focused on inorganic cathode materials with relatively limited specific capacities.^6–9^ Organic electrode materials derived from sustainable resources are promising candidates for the next-generation of energy storage materials.^10–15^ Different from inorganic materials containing expensive transition metals, organic compounds composed of C, H, and O are cost effective. Other advantages are the structural diversity and controllability of organic molecules, which can be designed to accommodate Na^+^ with high capacity through multi-electron reactions.

In our previous work, we reported a metal organic compound of cuprous tetracyanoquinodimethane (CuTCNQ) as a SIB cathode material showing a very high specific capacity of 252 mA h g^−1^.^16^ However, it was not practical since it did not contain active sodium. TCNQ is a redox-active molecule with a high redox potential and large capacity. Solid cells research and calculations have revealed it could work as a cathode with a two-electron reversible reaction.^17^ Even now, there are rarely reports of the cathode performance of TCNQ in organic electrolyte based rechargeable batteries, mainly due to its high solubility in the electrolyte. Here, we propose a simple strategy to allow the sodium salt of TCNQ (NaTCNQ) to work as a stable cathode material in SIBs, exhibiting a high specific capacity of 233 mA h g^−1^ at 20 mA g^−1^ with two discharge plateaus at 3.0 V and 2.2 V. NaTCNQ has an active sodium ion, so it can work with most sodium-poor anode materials. In this work, the NaTCNQ electrode shows a stable cycling performance for a highly reversible redox reaction by adding an effective adsorption layer composed of highly porous carbon material between the cathode and the separator, which prevents the dissolved organic compounds from moving to the anode side and protects the active material from loss. Furthermore, the electrochemical performance of NaTCNQ has been compared with some representative inorganic and organic cathode materials,^7–15^ which is shown in Fig. S1.† It is clear that NaTCNQ is a high performance organic cathode material combining high capacity with high potential.

## Experimental

### Materials synthesis

NaTCNQ was synthesized according to a solution reaction method similar to other metal-TCNQ compounds.^18^ Typically, 1.05 mmol NaI (99.99%, Aladdin) was dissolved in 25 mL degased acetonitrile (99.8%, SCR) to form solution A, while 1 mmol TCNQ (98%, Aladdin) was dissolved in 100 mL degased acetonitrile to form solution B. Then, solution A was injected into solution B rapidly under ultrasonic vibration. The yellow solution B instantly turned into a purple suspension and the mixture was maintained at room temperature (20 °C, RT) for 10 minutes. Then the formed dark purple powder was separated by filtration and washed with acetonitrile until the filtrate became colorless. Finally, the powder was dried at RT in air overnight.

### Preparation of the adsorption layer (AL)

Super P (SP) and activated carbon (AC) were selected as the composition materials of the adsorption layer. SP, or the mixture of SP and AC, was thoroughly ground. Then 80 wt% of carbon and 20 wt% of polyvinylidene fluoride (PVDF) were fully mixed in *N*-methyl pyrrolidone (NMP). Then, the slurry was uniformly coated onto a Celgard polypropylene (PP) membrane and dried at 80 °C for 12 h. Finally, the adsorption layer was cut into a wafer with a diameter of 19 mm.

### Materials characterization

XRD patterns were collected using a Panalytical X’pert PRO MRD (Holland) with Cu K_α_ radiation. A JSM 7600 FE-SEM (JEOL, Japan) was used to check the morphology and size of the materials. Brunauer–Emmett–Teller surface area measurements of the porous carbon were carried out with a volumetric sorption analyser (Beishide 3H-2000PM1, Beijing) using the physical adsorption and desorption of N_2_ at the liquid-nitrogen temperature. Pore size distributions were calculated according to the Barrett–Joyner–Halenda (BJH) method.

### Electrochemical characterization

The 2032 type coin cells were assembled and sealed in an Ar-filled glove box to study the electrochemical properties. The active electrodes were made by coating a slurry of 60 wt% NaTCNQ, 30 wt% Super P and 10 wt% PVDF onto carbon-coated aluminium foil. The electrodes were dried at 80 °C in an oven overnight and punched into round discs with a diameter of 8 mm. The typical mass loading of the electrodes was 0.80–1.2 mg cm^−2^. Sodium metal, a Whatman glass fiber membrane GF/B and 1 mol L^−1^ NaClO_4_ solution in EC/PC (1 : 1 v/v) were used as the counter electrode, separator and electrolyte, respectively. The adsorption layer was placed between the cathode and the separator. The charging–discharging cycles were performed on a battery measurement system (Land CT2001A, China) within the potential range 1.5–3.8 V (*vs.* Na^+^/Na) at RT. Electrochemical impedance spectra (EIS) measurements were recorded on a CHI660D (Shanghai, China) in the voltage range 1.5–3.8 V *vs.* Na^+^/Na and the frequency range 100 kHz to 0.1 Hz at RT.

## Results and discussion

### Structure and morphology of NaTCNQ

**Fig. 1:**
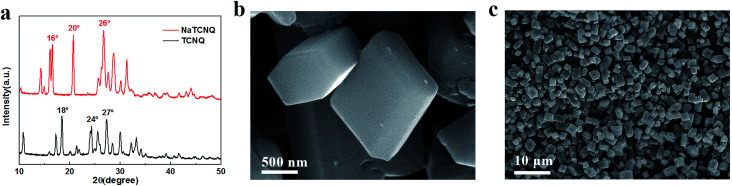
(a) XRD patterns of NaTCNQ and TCNQ (b) & (c) SEM images of the NaTCNQ powder.

The crystal structure of NaTCNQ was determined by X-ray powder diffraction in Fig. 1a. The main characteristic diffraction peaks of TCNQ are at 18°, 24° and 27°. After the reaction with NaI, the main peaks of the product emerge at 16°, 20° and 26°. The crystal structure of NaTCNQ was also investigated by Rietveld refinement of the powder XRD pattern according to previous literature.^19^ As shown in Fig. S2,† the refined structure fits well with the triclinic crystal system (*C*1̄ space group) with *R*_wp_ = 4.52% and *R*_p_ = 3.40%. The refined lattice parameters are: *a* = 6.986 Å, *b* = 23.643 Å, *c* = 12.441 Å, *α* = 90.140°, *β* = 98.580°, *γ* = 90.760° and *V* = 2023.868 Å^3^. The XRD refinement result confirms the conversion of TCNQ to the sodium salt (NaTCNQ). SEM images of the as-prepared NaTCNQ powder show a hexagonal-prism like morphology in Fig. 1b and c. The cross section is a regular symmetrical hexagon and the average size of the particles is approximately 1 μm.

### Electrochemical performance of NaTCNQ

We investigated the electrochemical performance of NaTCNQ as a cathode material for SIBs. We found it is not rechargeable in 1 M NaClO_4_ in EC/PC, as shown in Fig. S3a.† Associated with the solubility of TCNQ, this poor performance may be due to the dissolution of the active material. Then, we investigated the solubility of the NaTCNQ electrode, as displayed in Fig. S3b.† The color of the electrolyte rapidly changed to orange after the NaTCNQ electrode was immersed in the electrolyte, indicating that NaTCNQ is also easily soluble in the electrolyte. Since the separator is always porous and has little resistance to liquid penetration, the dissolved active material easily migrates through the separator to the anode side, resulting in extremely poor electrochemical properties.

**Fig. 2:**
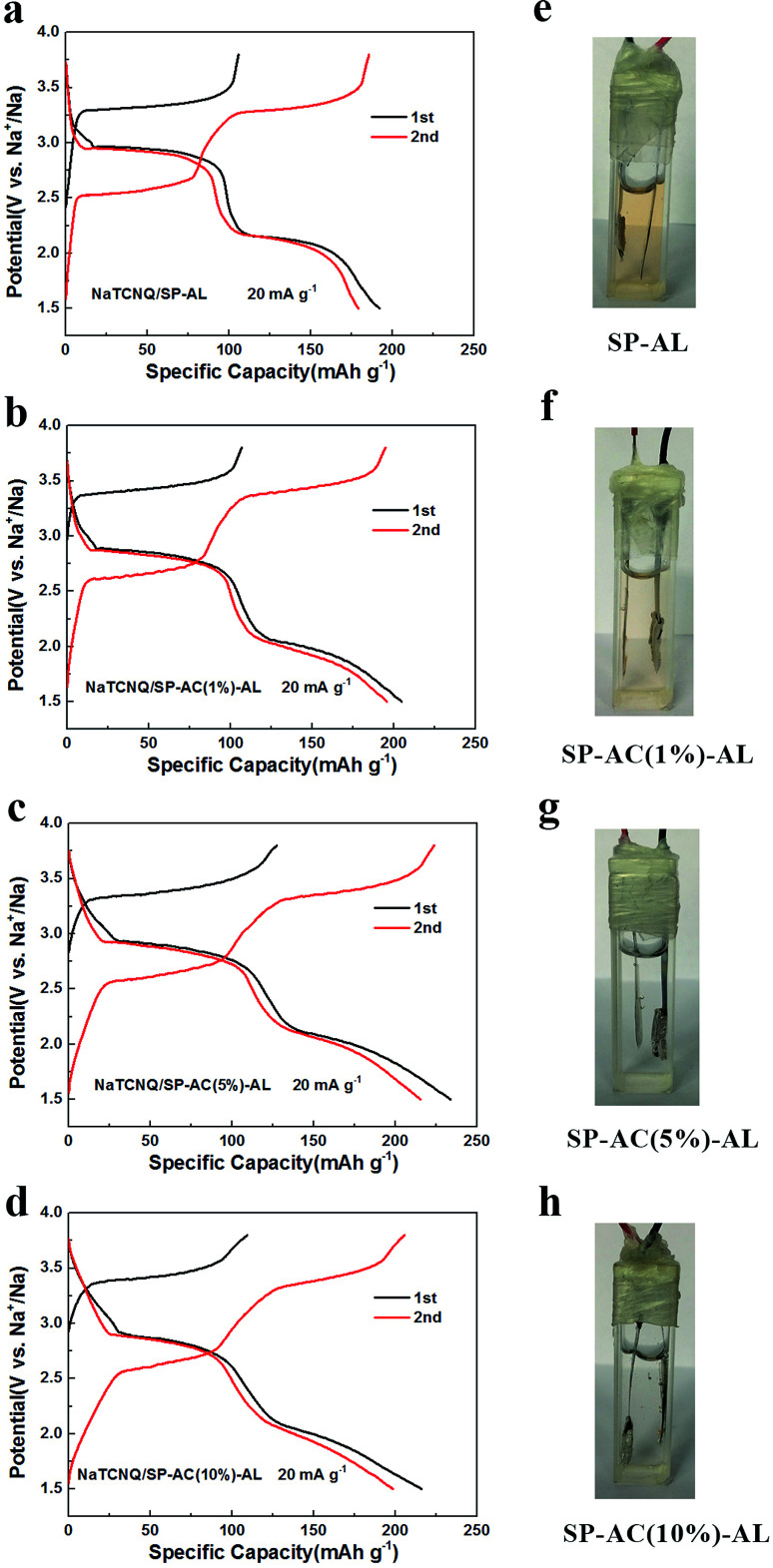
Charge discharge performance of (a) NaTCNQ/SP-AL (b) NaTCNQ/SP-AC (1%)-AL (c) NaTCNQ/SP-AC (5%)-AL and (d) NaTCNQ/SP-AC (10%)-AL at a current density of 20 mA g^−1^. Images of *in situ* cells of (e) NaTCNQ/SP-AL (f) NaTCNQ/SP-AC (1%)-AL (g) NaTCNQ/SP-AC (5%)-AL and (h) NaTCNQ/SP-AC (10%)-AL after two cycles at a current density of 20 mA g^−1^.

As reported in our previous study,^16^ a thin carbon film prepared from SP can be introduced as an adsorption layer (AL) to inhibit the shuttle effect of the dissolved active materials and serve as an upper current collector to increase active material utilization. It can significantly improve the cycling stability of organic electrode materials. We also studied the barrier effect of the Super P adsorption layer (SP-AL), as shown in Fig. 2a. It displays the initial two charge–discharge cycles within the voltage range 1.5–3.8 V at a current density of 20 mA g^−1^. In the first cycle, an evident charge voltage platform appears at 3.3 V and two distinct plateaus occur at 3.0 V and 2.2 V during discharging. It achieves a specific capacity of 192 mA h g^−1^ in the first discharging process, corresponding to 81% of the theoretical capacity of NaTCNQ (236 mA h g^−1^) calculated based on two electron transfer in the redox reaction of NaTCNQ. During the second cycle, both the charge and discharge processes present two potential plateaus, in accordance with a two-electron-transfer reaction (Fig. 2a). The corresponding redox reactions associated with the TCNQ species are illustrated in Scheme S1† according to a previous report.^17^ Meanwhile, the second discharge profile coincides well with the first cycle, suggesting that the redox reaction of NaTCNQ is reversible in the cell with an adsorption layer composed of Super P (SP-AL). To identify the adsorption effect of SP-AL, an operando battery was assembled in a quartz cell to observe the change in the electrolyte according to our previous report.^16^ As shown in Fig. 2e, after being charged and discharged for two cycles, the electrolyte in the cell with SP-AL is obviously discolored, but the color is much lighter than that in Fig. S3b,† demonstrating that the dissolution of the active material has been reduced. However, the color change of the electrolyte shows that SP-AL is not effective as a barrier to prevent the active material diffusing into the electrolyte. SP is a porous and superior conductive carbon with a surface area of about 75 m^2^ g^−1^, which is rather limiting for effective adsorption. AC is extremely porous and has a very large surface area higher than 1000 m^2^ g^−1^ with the strongest physical adsorption capacity. The great interfacial tension of pristine AC makes it hard to form a uniform adhesive film on the PP membrane, even when the AC content is more than 10%. Finally, we selected three different AC contents of 1%, 5% and 10% to prepare the carbon AL. Fig. 2b–d displays the charge–discharge curves of the NaTCNQ cells assembled with SP-AC (1%)-AL, SP-AC (5%)-AL and SP-AC (10%)-AL within the voltage range 1.5–3.8 V at a current density of 20 mA g^−1^. All three samples exhibit similar charge and discharge profiles, which are less flat than that of NaTCNQ/SP-AL because AC has capacity contributions in the sodium ion battery. As shown in Fig. S4,† AC gives a discharge capacity of approximately 80 mA h g^−1^ in the voltage window 1.5–3.8 V *vs.* Na^+^/Na with a slope voltage profile. However, when the AC content is reduced to 1%, 5% and 10%, the discharge capacity is decreased to 9.3, 10.2 and 12.8 mA h g^−1^. The initial discharge capacity of NaTCNQ/SP-AC (1%)-AL, NaTCNQ/SP-AC (5%)-AL and NaTCNQ/SP-AC (10%)-AL is 204 mA h g^−1^, 233 mA h g^−1^ and 216 mA h g^−1^, respectively, which amount to 86%, 98% and 91% of the theoretical capacity. Moreover, the electrolyte in the operando cell with SP-AC (1%)-AL is slightly discolored (Fig. 2f), but the other two with a higher AC content are still unchanged and clear after two cycles (Fig. 2g and h), implying that the AC-containing AL is effective at stopping organic active materials dissolving into the electrolyte.

**Fig. 3:**
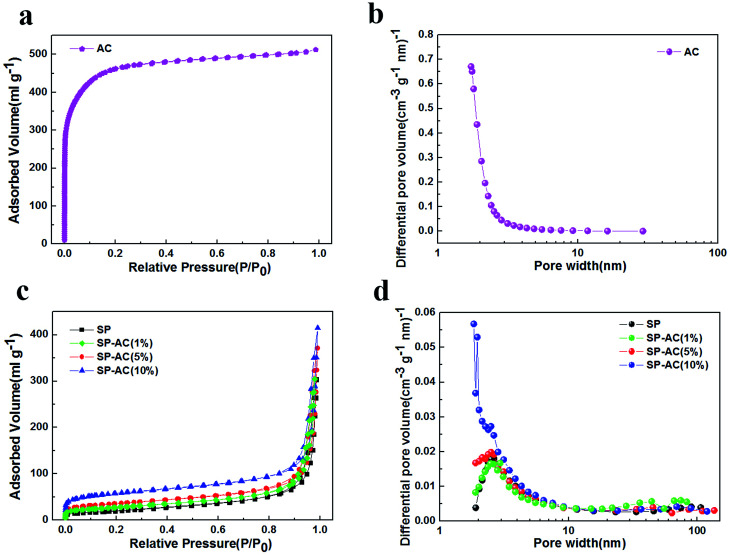
(a) & (c) Nitrogen adsorption isotherms and (b) & (d) pore size distributions (BJH method) of AC, SP, SP-AC (1%), SP-AC (5%), and SP-AC (10%).

Specific surface area (SSA) and pore size are important parameters in adsorptive materials,^20,21^ therefore we measured the BET curves and pore size distribution plots of SP, SP-AC (1%), SP-AC (5%), SP-AC (10%) and the AC powder in Fig. 3 and the results obtained by the fitting calculation are displayed in Table S1.† It is clear that the AC powder has a super high surface area of 1592 m^2^ g^−1^, which is 20 times higher than that of SP, indicating that AC can provide many more active adsorption sites. Even with the low AC content of SP-AC (5%) and SP-AC (10%), the specific surface areas are greatly improved associated with enhanced physisorption capability. Although the nitrogen adsorption isotherms and pore size distributions suggest micropores (<2 nm) are dominant in the AC powder, the low content of AC (<10%) does not change the typical capillary condensation of mesopores (2–50 nm) in SP (Fig. 3c, d).

**Fig. 4:**
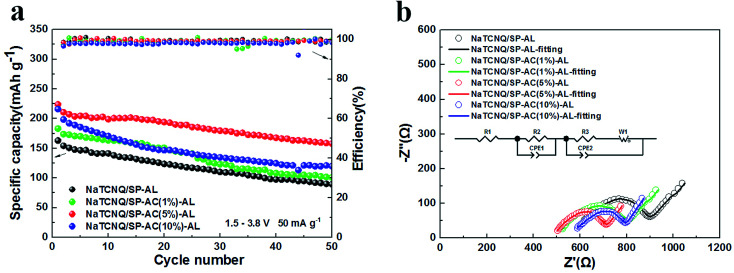
(a) Cycling performance of NaTCNQ/SP-AL, NaTCNQ/SP-AC (1%)-AL, NaTCNQ/SP-AC (5%)-AL and NaTCNQ/SP-AC (10%)-AL electrodes in the voltage range of 1.5–3.8 V (*vs.* Na^+^/Na) at 50 mA g^−1^. (b) Electrochemical Impedance Spectroscopy (EIS) of NaTCNQ/SP-AL, NaTCNQ/SP-AC (1%)-AL, NaTCNQ/SP-AC (5%)-AL and NaTCNQ/SP-AC (10%)-AL electrodes in the frequency range 100 kHz to 0.1 Hz. Inset: equivalent circuit of the EIS curves.

Since the AC-containing carbon powders have higher physisorption capability than the pristine SP powder, we further investigated the rechargeability of the NaTCNQ cells assembled with different carbon ALs. Fig. 4a exhibits the cycling performance of the NaTCNQ cells with various adsorption layers at a current density of 50 mA g^−1^. For the NaTCNQ/SP-AL cell, the initial discharge specific capacity is 163 mA h g^−1^ and it is reduced to 90 mA h g^−1^ after 50 cycles, showing 55% capacity retention. The first discharge capacity of the NaTCNQ/SP-AC (1%)-AL, NaTCNQ/SP-AC (5%)-AL and NaTCNQ/SP-AC (10%)-AL cells is 183 mA h g^−1^, 224 mA h g^−1^ and 216 mA h g^−1^, respectively. After 50 cycles, the capacity retention is 55%, 70% and 55%. These results demonstrate the significantly improved cycling stability of the NaTCNQ cells with carbon adsorption layers, which enables the reversible redox reaction of the TCNQ species in liquid electrolyte sodium ion batteries. Especially in the NaTCNQ cell with SP-AC (5%)-AL, high physisorption further provides increased capacity and improved cycling performance.

To gain further insight into the differences in the electrochemical properties of the various adsorption layers, electrochemical impedance spectroscopy (EIS) of the cells with SP-AL, SP-AC (1%)-AL, SP-AC (5%)-AL and SP-AC (10%)-AL was performed. The EIS curves consist of a depressed semicircle in the high frequency region and an inclined line in the low frequency region, and we fitted the curves as displayed in Fig. 4b. The Fig. 4b inset shows the equivalent circuit of the cells. *R*_1_ stands for the electrolyte resistance, *R*_2_ is the interface resistance for the carbon adsorption layer and *R*_3_ corresponds to the electrochemical reaction resistance in the NaTCNQ electrode. *R*_w_ is the Warburg impedance and is related to the diffusion of Na^+^ within the electrode. Table 1 shows the fitted resistance values of the cells. We found that all of the *R*_1_ values are large, which is ascribed to the poor wettability of the Celgard membrane in the cyclic carbonate-based electrolyte. This is the reason why NaTCNQ batteries exhibit much lower capacities at a little higher current density. Also, it is clear that the electrolyte resistance and the interface resistance reduce firstly with the addition of AC, which is due to the better infiltration of the electrolyte in the highly porous carbon, and it is lowest when the content of AC is 5%. When more AC is added up to 10%, *R*_1_ and *R*_2_ appear to increase owing to the poor contact of AC with the PP film due to its much higher surface tension. The EIS results clearly show that the NaTCNQ/SP-AC (5%)-AL cell achieved the lowest impedance and greatly improved the electrochemical performance.

**Table 1:** The fitted resistance values of the NaTCNQ/SP-AL, NaTCNQ/SP-AC (1%)-AL, NaTCNQ/SP-AC (5%)-AL and NaTCNQ/SP-AC (10%)-AL cells

Sample	*R*_1_ (ohm)	*R*_2_ (ohm)	*R*_3_ (ohm)	*R*_w_ (ohm)
NaTCNQ/SP-AL	578.2	142.1	138.5	21.7
NaTCNQ/SP-AC (1%)-AL	512.1	136.5	131.8	20.6
NaTCNQ/SP-AC (5%)-AL	492.7	71.7	137.0	17.8
NaTCNQ/SP-AC (10%)-AL	565.9	104.6	120.6	18.9

## Conclusions

In summary, we have successfully synthesized the redox-active organic salt NaTCNQ. However, the NaTCNQ electrode shows non-rechargeability due to the high solubility of the active material in the carbonate-based liquid electrolyte. By introducing a porous carbon adsorption layer between the organic electrode and the separator, the migration of dissolved active material is suppressed. An adsorption layer with higher physisorption capability is obtained by adding a low content of high-surface-area activated carbon powder, which can significantly improve the reversibility of the redox reaction of the NaTCNQ electrode. Combining higher adsorption and better infiltration, the NaTCNQ/SP-AC (5%)-AL cell displays a high initial capacity of 233 mA h g^−1^ (98.7% of the theoretical value) at 20 mA g^−1^ and exhibits 70% capacity retention after 50 cycles at 50 mA g^−1^. Our work provides a facile method to enable soluble organic electrodes to be utilized as highly reversible electrode materials for high performance lithium/sodium ion batteries.

## Conflicts of interest

There are no conflicts to declare.

## Supplementary Material

RA-008-C8RA03093F-s001
