# Supplementary material for: Porous carbon adsorption layer enabling highly reversible redox-reaction of a high potential organic electrode material for sodium ion batteries
Source: RSC Adv. 2018 Jul 11;8(44):24900–5. doi: 10.1039/c8ra03093f (PMC9082292; doi:10.1039/c8ra03093f)
Supplement: RA-008-C8RA03093F-s001 [file RA-008-C8RA03093F-s001.pdf]

Electronic Supplementary Material (ESI) for RSC Advances.
This journal is © The Royal Society of Chemistry 2018

Supporting Information

Porous Carbon Adsorption Layer Enabling Highly Reversible Redox-Reaction of High Potential Organic Electrode Material for Sodium Ion Batteries

Yanjie Wang,[a] Chun Fang,*[b] Ying Huang,[c] Qing Liu,[b] Ruirui Zhao,[a] Xuli Ding [ad] and Yunhui Huang *[ab]

a.Collaborative Innovation Center of Intelligent New Energy Vehicle, School of Materials Science and Engineering Tongji University, Shanghai 201804, China.E-mail: huangyh@tongji.edu.cn
b.State Key Laboratory of Material Processing and Die & Mould Technology, School of Materials Science and Engineering Huazhong University of Science and Technology,Wuhan, Hubei 430074, China. E-mail: fangchun@hust.edu.cn
c.School of Materials Science and Engineering, Yunnan Key Laboratory for Micro/Nano Materials & Technology Yunnan University Kunming, Yunnan 650091, China.
d.Department of Physics, Jiangsu University of Science and Technology, Zhenjiang, 212003, China.

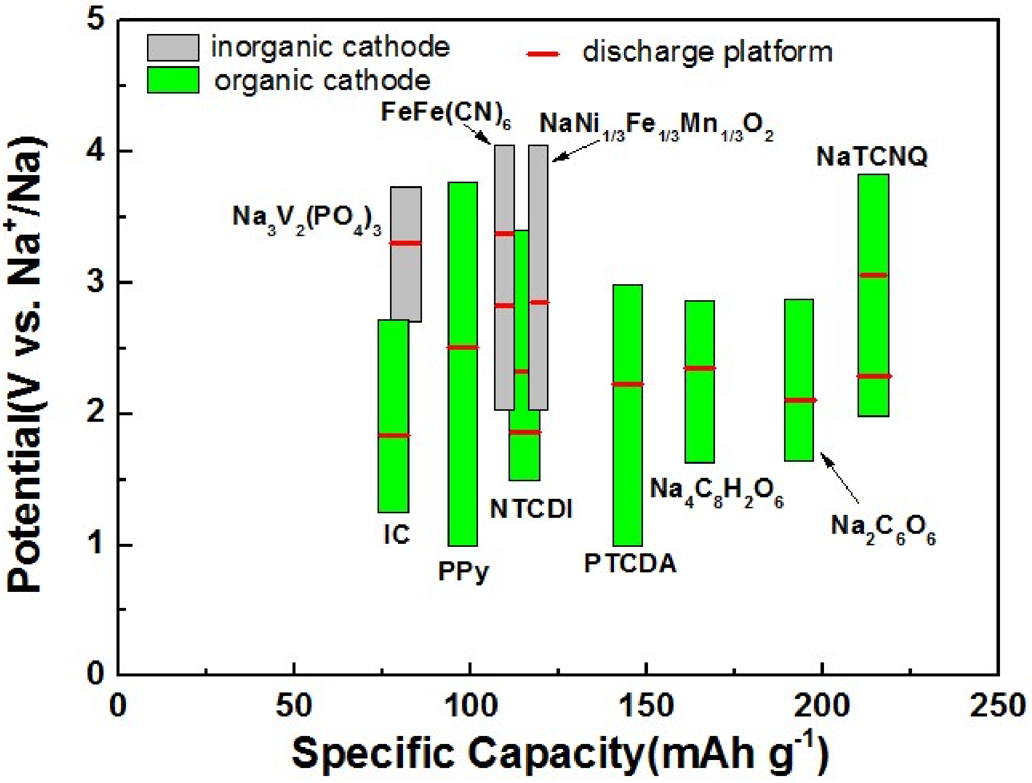

Fig S1. The electrochemical properties of some representative cathode materials, including operating voltage range and discharge platform(versus Na^+/Na) and specific capacity. ($NaNi_{1/3}Fe_{1/3}Mn_{1/3}O_2$ [7], $Na_3V(PO_4)_3$ [8], $FeFe(CN)_6$ [9], indigo carmine(IC) [10], polypyrrole(PPy) [11], 1,4,5,8-naphthalenetetracarboxylic diimide (NTCDI) [12], 3,4,9,10-perylene-tetracarboxylicacid-dianhydride (PTCDA) [13], tetrasodium salt of 2,5-dihydroxyterephthalic acid ($Na_4C_8H_2O_6$) [14], $Na_2C_6O_6$ [15])

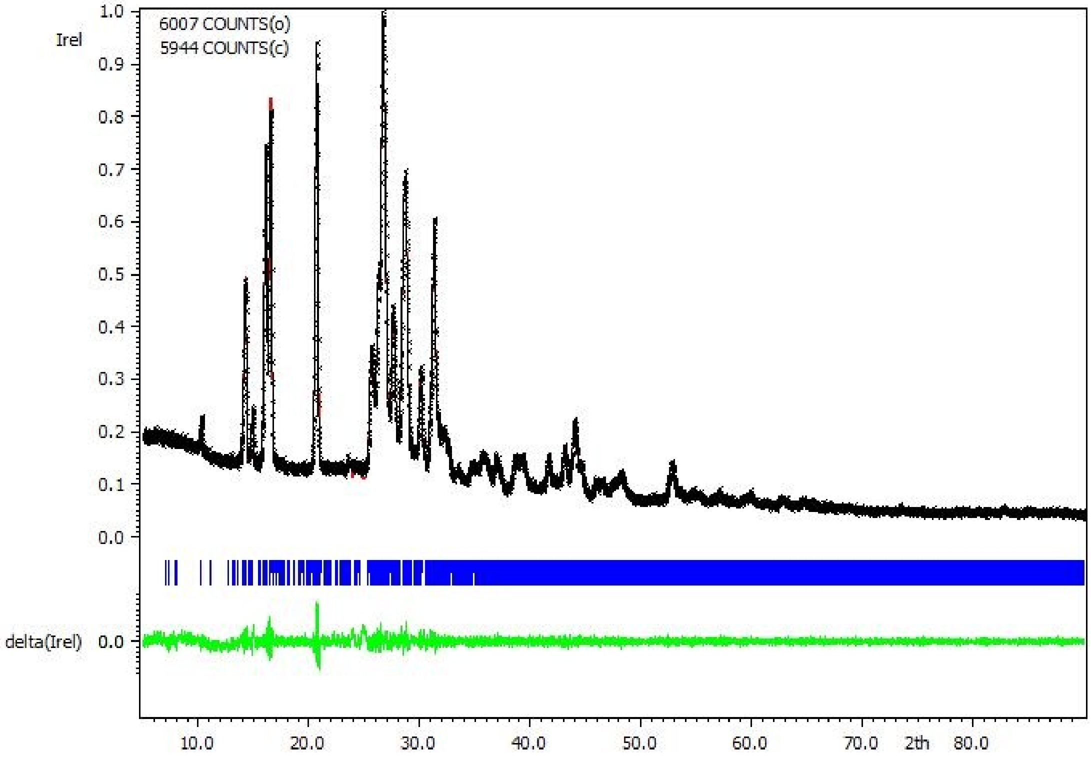

Fig. S2. The XRD profile Rietveld refinement of NaTCNQ powder.

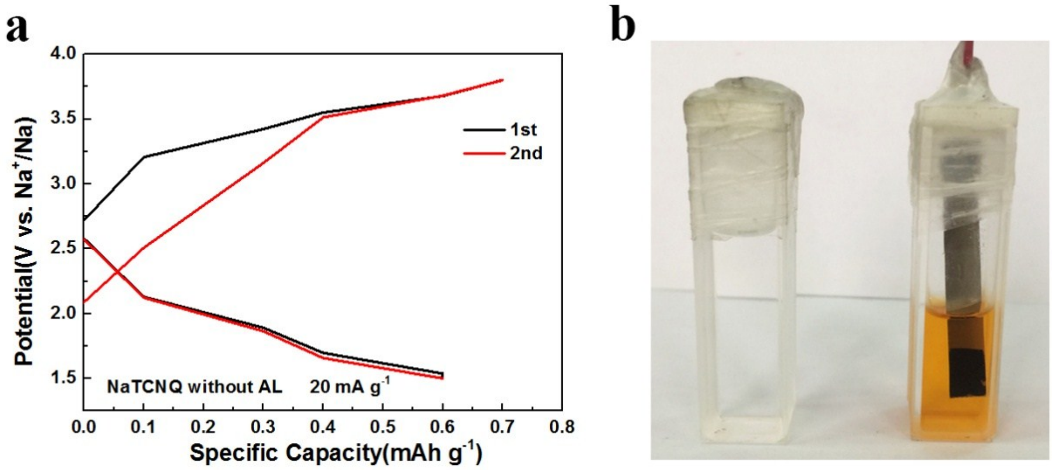

Fig. S3. (a) Charge discharge performance of NaTCNQ without AL at a current density of 20 mA g^{-1}. (b) The 1 M $NaClO_4$ in EC/PC (1:1 v:v) electrolyte (left) and the dissolution behavior of NaTCNQ electrode in it (right).

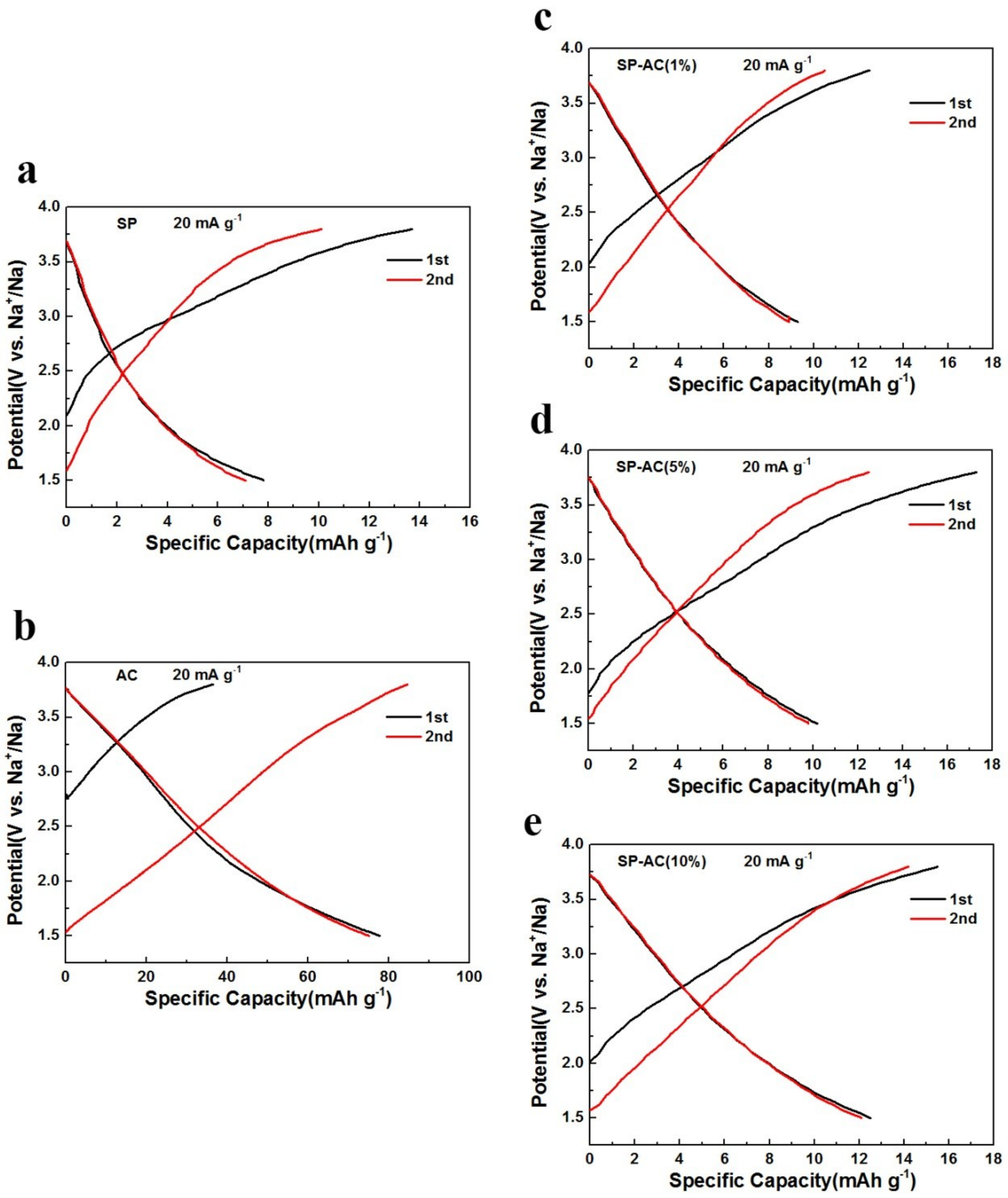

Fig. S4. Charge discharge performance of (a) SP (b) AC (c) SP-AC (1%) (d) SP-AC (5%) (e) SP-AC (10%) at a current density of 20 mA g^{-1}.

In the charge process

The first cycle

$$NaTCNQ - e^- \rightarrow TCNQ^0 + Na^+ \text{ (OCV - 3.8 V)} \quad (1)$$

After the first cycle

$$[Na(TCNQ)^{2-}]^- - e^- \rightarrow NaTCNQ \text{ (1.5 - 3.0 V)} \quad (2)$$

$$NaTCNQ - e^- \rightarrow TCNQ^0 + Na^+ \text{ (3.0 - 3.8 V)} \quad (3)$$

In the discharge process

$$TCNQ^0 + Na^+ + e^- \rightarrow NaTCNQ \text{ (3.8 - 3.0 V)} \quad (4)$$

$$NaTCNQ + e^- \rightarrow [Na(TCNQ)^{2-}]^- \text{ (3.0 - 1.5 V)} \quad (5)$$

Scheme S1. Charge and discharge equations of NaTCNQ

Table S1. Specific surface area (SSA), and most probable pore diameter (MPPD) of SP, SP-AC (5%), SP-AC (10%) and AC by fitting calculation.

Carbon	SSA/m^2 g^{-1}	MPPD/nm
SP	75	2.56
SP-AC (1%)	97	2.42
SP-AC (5%)	122	2.43
SP-AC (10%)	196	1.82
AC	1592	1.71

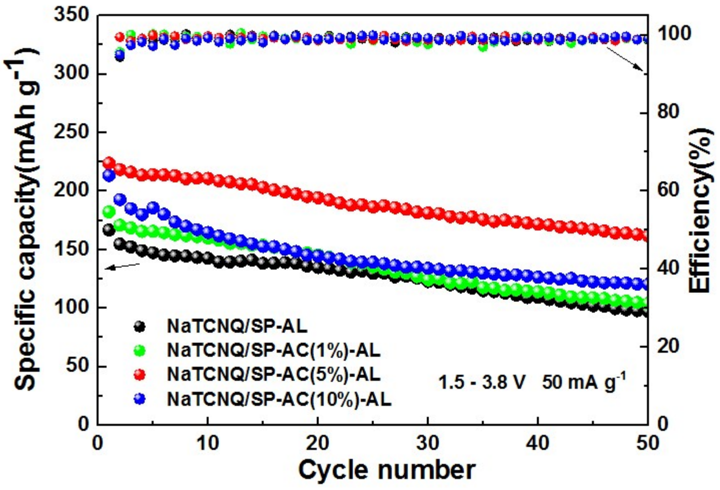

Fig. S5. Cycling performance of NaTCNQ/SP-AL, NaTCNQ/SP-AC (1%)-AL, NaTCNQ/SP-AC (5%)-AL and NaTCNQ/SP-AC (10%)-AL electrodes in the voltage range of 1.5-3.8 V (vs. Na$^+$/Na) at 50 mA g^{-1} (repeatable data).
